# Recognition of ASF1 by Using Hydrocarbon‐Constrained Peptides

**DOI:** 10.1002/cbic.201800633

**Published:** 2019-02-13

**Authors:** May Bakail, Silvia Rodriguez‐Marin, Zsófia Hegedüs, Marie E. Perrin, Françoise Ochsenbein, Andrew J. Wilson

**Affiliations:** ^1^ Institute for Integrative Biology of the Cell (I2BC) IBITECS CEA CNRS Université Paris–Sud Université Paris–Saclay 91198 Gif-sur-Yvette Cedex France; ^2^ Present address: Inserm, U1016 Institut Cochin CNRS UMR8104 Université Paris Descartes 27, rue du Faubourg Saint-Jacques 75014 Paris France; ^3^ School of Chemistry University of Leeds Woodhouse Lane Leeds LS2 9JT UK; ^4^ Astbury Centre for Structural Molecular Biology University of Leeds Woodhouse Lane Leeds LS2 9JT UK

**Keywords:** chemical biology, constrained peptides, histone chaperones, protein surface recognition, protein–protein interactions

## Abstract

Inhibiting the histone H3–ASF1 (anti‐silencing function 1) protein–protein interaction (PPI) represents a potential approach for treating numerous cancers. As an α‐helix‐mediated PPI, constraining the key histone H3 helix (residues 118–135) is a strategy through which chemical probes might be elaborated to test this hypothesis. In this work, variant H3_118–135_ peptides bearing pentenylglycine residues at the *i* and *i*+4 positions were constrained by olefin metathesis. Biophysical analyses revealed that promotion of a bioactive helical conformation depends on the position at which the constraint is introduced, but that the potency of binding towards ASF1 is unaffected by the constraint and instead that enthalpy–entropy compensation occurs.

A significant unmet goal in chemical biology is to develop methods for inhibiting protein–protein interactions (PPIs).^1^, ^2^ In the context of α‐helix‐mediated PPIs,^3^ considerable effort has been exerted on developing methods for constraining (or “stapling”) peptides in an α‐helical conformation. This approach has been used to confer enhanced proteolytic stability, enhanced cell‐uptake and, in some cases, enhanced target affinity on constrained peptide sequences.^4^, ^5^, ^6^, ^7^, ^8^, ^9^, ^10^, ^11^, ^12^, ^13^, ^14^, ^15^, ^16^, ^17^, ^18^, ^19^, ^20^, ^21^, ^22^, ^23^ We recently introduced a series of reagents and approaches for constraining peptides in a helical conformation.^24^, ^25^, ^26^, ^27^ Of these, the use of variant peptides bearing alkenyl glycine residues in the *i* and *i*+4 positions constrained through olefin metathesis was shown to be effective in biasing the sequences of variant BCL‐2 BH3 sequences towards the helical conformation.^27^ Subsequently, we demonstrated that these peptides bind to their target BCL‐2 proteins through an induced‐fit mechanism but do not elicit enhanced target affinity arising from enthalpy–entropy compensation, as demonstrated by surface plasmon resonance (SPR) and van't Hoff analyses, respectively.^26^ Herein, using the anti‐silencing function 1 (ASF1) chaperone as a protein target, we demonstrate the broader applicability of *S*‐pentenyl‐glycine variant peptides as substrates for hydrocarbon constraining and further reinforce the notion that constraining the peptide in a bioactive conformation might not lead to increased affinity for the target protein due to enthalpy–entropy compensation.

Histone chaperones regulate the association of basic histone proteins with DNA, thereby permitting nucleosome assembly in an ordered and controlled manner.^28^, ^29^, ^30^, ^31^, ^32^, ^33^ ASF1 is a highly conserved histone chaperone that is involved in both histone H3–H4 handling and buffering.^34^, ^35^, ^36^, ^37^, ^38^ It has been shown to play a key role in the development and progression of some cancers; hence, it is a potential target for chemical probes and drug discovery.^39^, ^40^, ^41^ The interaction between ASF1 and the H3 and H4 histone proteins forms a ASF1–(H3–H4) complex that prevents the formation of the histone H3–H4 tetramer and shields H3–H4 dimers from unfavourable interactions. Re‐establishment of the tetramer was proposed to be the key element for the formation of the nucleosome (Figure 1 A).^42^ The ASF1 protein comprises a conserved N‐terminal domain of 156 amino acids, which is essential for its function in vivo, and a divergent unstructured C‐terminal domain, which is not considered necessary for function.^37^, ^43^ Its structure comprises an elongated β sandwich core with three α‐helices in the loops between the β‐strands (Figure 1 B). The contacts between H3 and ASF1 are extensive and result in a buried surface area of 909 Å^2^. The histone H3 binding site is located in the concave face of ASF1 (Figure 1 B) and involves β‐strands β3, β4 and β6–9.^37^, ^43^, ^44^ The main interactions occur through the C‐terminal helix of H3 (residues 122–134), where the key residues Leu126 and Ile130 form a hydrophobic clamp with the hydrophobic region of ASF1. Additionally, there is a network of electrostatic interactions at the PPI interface, such as the salt bridge between Arg129 from H3 and Asp54 from Asf1.^45^ The ASF1–H3–H4 structure also shows extensive contacts between ASF1 and histone H4^44^ in two parts (not shown): the globular core of ASF1 interacts with the C‐terminal tail of H4 to form a strand‐swapped dimer, and the C‐terminal tail of ASF1 binds to the histone fold region of histone H4.

**Figure 1:**
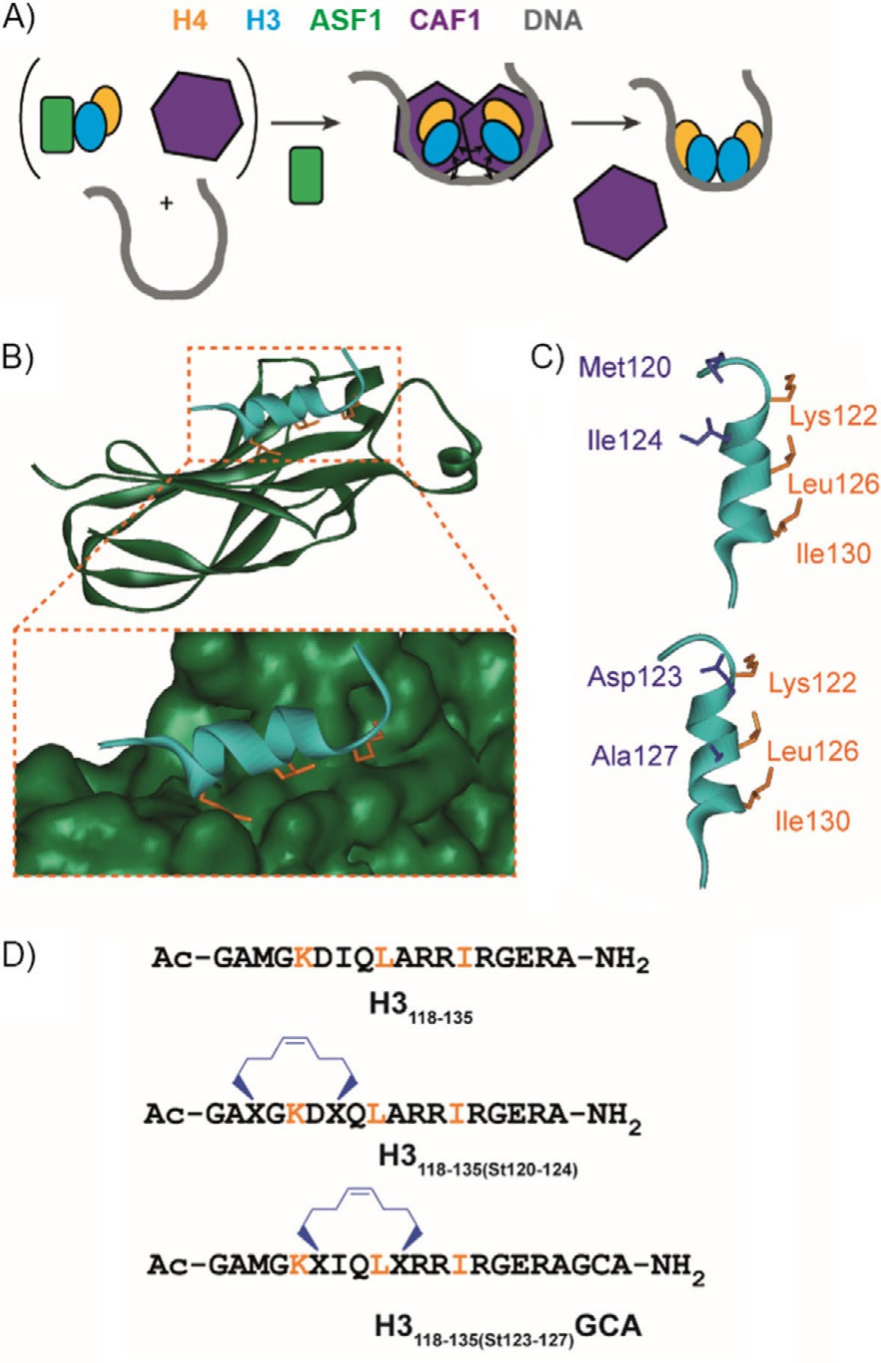
ASF1 as a target for constrained peptides. A) Schematic illustration of the role of ASF1 (green) in displacing CAF‐1 (purple) through the recognition of histone H3 (cyan) and H4 (yellow) so as to facilitate nucleosome formation. B) Structure of the histone H3(118–135) (cyan)–ASF1A(1–156) (dark green) interaction as determined by NMR spectroscopy (PDB ID: 2IIJ)^[45]^—the histone side chains located on one face that are perceived to be important for binding are shown as orange sticks. C) The key H3 helix (cyan), key side chains (orange) and residues at *i*, *i*+4 positions considered suitable for introduction of a constraint (purple) are highlighted. D) Sequences of the peptides used in this study with the positions of the hydrocarbon constraints.

We envisioned the C‐terminal α‐helix peptide of H3 as a template for the design of molecules able to recognise ASF1. We used *S*‐pentenylglycine rather than *S*‐pentenylalanine, as the former is easier to synthesise and demonstrates comparable behaviour in biophysical analyses.^27^ The sites to incorporate the mono‐alkenyl‐substituted amino acids within the peptide sequence were selected by taking into account: 1) the requirement to appropriately position the unnatural amino acids so as to constrain in a manner that promotes a helical conformation (i.e., the *i* and *i*+4 positions); 2) the need to position the hydrocarbon bridge so as not to sterically occlude the “wild‐type” interactions necessary for recognition. On this basis, two options were considered Met120/Ile124 and Asp123/Ala127. H3_118–135_, together with variants bearing *S*‐pentenylglycine in the identified positions were prepared by solid‐phase peptide synthesis (see the Supporting Information), and the latter were crosslinked by olefin metathesis to give H3_118–135(St120–124)_ and H3_118–135(St123–127)_GCA (the GCA sequence was introduced for future functionalisation, e.g., cell‐penetrating sequences, fluorophores, etc. through the nucleophilic thiol of the cysteine residue). On‐resin ring closure proceeded quantitatively in 4 h.

The helical character of all three peptides was investigated by using circular dichroism (CD) in both 40 mm phosphate buffer and trifluoroethanol (TFE). In aqueous solvent, H3_118–135_ and H3_118–135(St120–124)_ both gave CD spectra consistent with a predominantly random‐coil conformation (% helicities H3_118–135_=15 % and H3_118–135(St120–124)_=20 % ), whereas in the presence of the helix‐promoting TFE (Figure S1 in the Supporting Information)^46^, ^47^ the CD spectra were indicative of a more α‐helical signature, thus indicating that both possess sufficient conformational flexibility to access the helical conformation required for specific ASF1 binding. It is perhaps unsurprising that constraining the peptide between residues 120 and 124 did not promote a helical conformation in H3_118–135(St120–124)_ given the observation from the H3/ASF1 NMR structure that the H3 helix is distorted/frayed at the N terminus close to M120. In contrast, CD analyses showed H3_118–135(St123–127)_GCA to adopt a more helical conformation in aqueous solution by (% helicity=29 %), as expected. The data for all three peptides in TFE (see the Supporting Information) demonstrate that each is capable of adopting a helical conformation to a comparable extent, and, that there is little difference between buffer and TFE for H3_118–135(St123–127)_GCA; this indicates that the sequence has intrinsically low helical propensity. (Figure 2).

**Figure 2:**
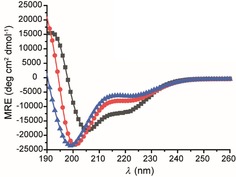
Conformation analyses of histone H3 variant peptides ▴: H3_118–135_, •: H3_118–135(St120–124)_ and ▪: H3_118–135(St123–127)_GCA (100 μm in 40 mm sodium phosphate, pH 7.5, 293 K) by CD analyses.

Binding of the peptides to ASF1 was then assessed by isothermal titration calorimetry (ITC; Figure 3, Table 1). All three peptides exhibited exothermic binding and could be fitted to a 1:1 binding isotherm. Strikingly, the binding potency was similar in all three cases, Δ*G*=−7.3 to −8.0 kcal m ^−1^, despite H3_118–135(St123–127)_GCA adopting a more helical conformation and therefore presumably being more pre‐organised towards ASF1 recognition. Analyses of the thermodynamic determinants of binding reveal enthalpy–entropy compensation. Both H3_118–135_ and H3_118–135(St120–124)_ exhibited favourable enthalpies of binding (Δ*H*=−14.4 to −15.0 kcal m ^−1^) but the binding entropies were unfavourable (*T*Δ*S*=−6.4 to −6.7 kcal m ^−1^). In contrast, for the more helical peptide, H3_118–135(St123–127)_GCA, the entropy of binding (*T*Δ*S*=3.1 kcal m ^−1^) was favourable, consistent with the anticipated effect of pre‐organisation; however, the enthalpy of binding (Δ*H*=−4.2 kcal m ^−1^) was less favourable than for the less helical variants. It is noteworthy that, despite the fact that both contain a staple, H3_118–135(St120–124)_ is less helical and exhibits a large favourable enthalpy change with unfavourable entropy, whereas H3_118–135(St123–127)_GCA is more helical and has a less favourable enthalpy of binding but more favourable entropy of binding. Such an effect might arise because the less helical peptides H3_118–135_ and H3_118–135(St120–124)_ form enthalpically favourable backbone hydrogen bonds upon a change in conformation to the helix, whereas the more pre‐organised helix H3_118–135(St123–127)_GCA neither gains new hydrogen bonds nor undergoes an entropically costly change in conformation on binding ASF1. Alternatively, differential changes in the solvation of the peptides upon binding could account for such a difference in thermodynamic signature. Either way, the results underscore a limitation in correlating conformational stability against binding potency for the unconstrained (H3_118–135_) and constrained (H3_118–135(St123–127)_GCA) peptides; although the helical conformation is preferred for H3_118–135(St123–127)_GCA, this can be considered as arising from an increase in energy (or destabilisation) of nonhelical conformations for this sequence as opposed to preorganisation of the wild‐type sequence.

**Figure 3:**
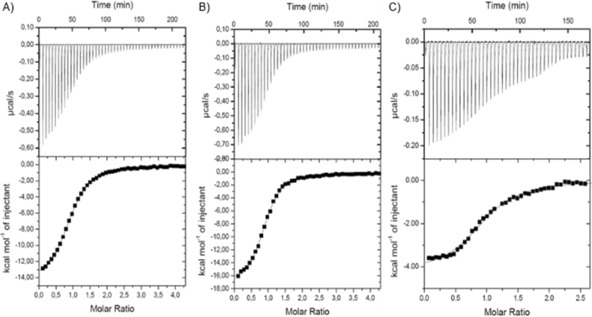
ITC thermograms and data fitting for the interaction of A) H3_118–135_, B) H3_118–135(St120–124)_ and C) H3_118–135(St123–127)_GCA with ASF1A(1–156).

**Table 1:** Thermodynamic parameters for the binding of histone H3 peptide variants to ASF1 as determined by ITC (see Figure 3 for details)

Peptide	*K*_d_ [μm]	Δ*G* [kcal m ^−1^]	N*	Δ*H* [kcal m ^−1^]	*T*Δ*S* [kcal m ^−1^]
H3_118–135_	1.34±0.33	−8.0±0.14	0.94±0.04	−14.4±0.37	−6.4±0.51
H3_118–135(St120–124)_	0.86±0.11	−8.3±0.07	0.97±0.01	−15±0.96	−6.7±1.03
H3_118–135(St123–127)_GCA	1.6±0.13	−7.3±0.05	0.97±0.01	−4.2±0.08	3.1±0.2

In order to confirm the binding mode of the constrained peptides with ASF1, chemical‐shift‐perturbation studies were carried out for all three peptides (Figure S2) by using uniformly ^15^N labelled ASF1A(1–156). The chemical‐shift variation was mapped onto the protein structure of ASF1A–H3 (PDB ID: 2IIJ). All three peptides induced the highest values of chemical‐shift variation and a “slow‐exchange” regime for the ASF1 residues defining the already well characterised H3 binding site (V45–E51, V90–I97, R108–Y111, V146–T147),^37^, ^45^ thus confirming the preservation of the specific binding mode for the constrained peptides. In addition, both H3_118–135_ and H3_118–135(St120–124)_ exhibited chemical‐shift variations on the opposite side of the protein surface corresponding to the B domain binding site (S59–F72);^48^ these most probably correspond to nonspecific binding in the case of the histone peptide. Interestingly, constrained H3_118–135(St123–127)_GCA induced no chemical‐shift variation in this region of ASF1 (Figure S2 B). This result suggests that unfolding of the helical conformation is probably required for this nonspecific binding.

The proteolytic stability of the peptides was also investigated by using trypsin and proteinase K. The unconstrained H3_118–135_ was cleaved within 14 minutes by both proteases (Figure 4, Table 2 and the Supporting Information), whereas the constrained peptides had increased stability depending on the position of the constraint. H3_118–135(St120–124)_ was less susceptible to cleavage by proteinase K (*t*_1/2_=65.8 min). On the other hand H3_118–135(St123–127)_GCA showed increased stability against trypsin (*t*_*1*/2_=40.5 min). The constraint also affected the profile of cleavage sites, most notably for H3_118–135(St123–127)_GCA, for which two proteinase K cleavage sites were suppressed by introducing the constraint. Thus, the results of proteolytic cleavage studies on constrained peptides need to be considered, as the protective effect is likely to arise not only from the enhanced helicity, that is, the greatest effect is observed for the constraint that does not markedly promote helicity (H3_118–135(St120–124)_).

**Figure 4:**
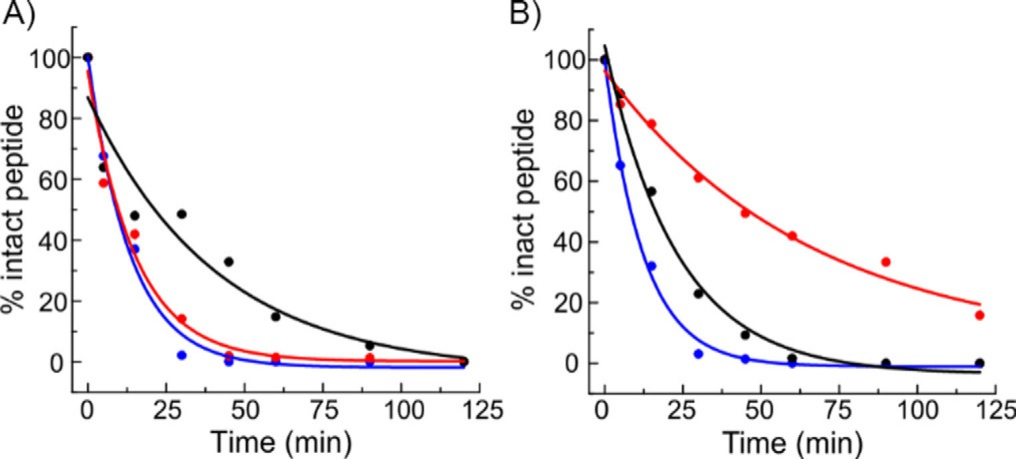
Proteolytic stability of peptides ▴: H3_118–135_, •: H3_118–135(St120–124)_ and ▪: H3_118–135(St123–127)_GCA against A) trypsin and B) proteinase K.

**Table 2:** Fitted half‐lives of the peptides in the presence of proteases.

Peptide	Trypsin *t*_1/2_ [min]	Proteinase K *t*_1/2_ [min]
H3_118–135_	13.3±1.5	12.2±0.8
H3_118–135(St120–124)_	14.9±2.5	65.8±15.7
H3_118–135(St123–127)_GCA	40.5±16.9	23.1±2.6

In conclusion, we have shown that variant H3_118–135_ peptides with pentenylglycine residues at the *i* and *i*+4 positions can be constrained by olefin metathesis to generate a peptide more biased towards a helical conformation than the parent sequence, thus further broadening the scope of this unnatural amino acid for hydrocarbon “stapling”. In addition, we have illustrated that a more helical conformation (i.e., for H3_118–135(St123–127)_GCA) does not necessarily correlate with significant proteolytic protection or enhanced binding potency; rather where the later aspect is concerned, enthalpy–entropy compensation is observed. Nonetheless, constraining peptides has been shown to reduce nonspecific binding and to enhance a range of additional pharmacokinetic properties such as cellular uptake. The peptide sequence used in this work was shown to have moderate helical propensity. Thus our future studies will centre on exploiting the constraining strategy together with helix‐stabilising amino acids to optimise these reagents for binding and cell permeability so as to develop chemical probes of the H3‐ASF1 interaction.

## Conflict of interest

*The authors declare no conflict of interest*.

## Supporting information

As a service to our authors and readers, this journal provides supporting information supplied by the authors. Such materials are peer reviewed and may be re‐organized for online delivery, but are not copy‐edited or typeset. Technical support issues arising from supporting information (other than missing files) should be addressed to the authors.

Supplementary
