# Supplementary material for: Recognition of ASF1 by Using Hydrocarbon‐Constrained Peptides
Source: Chembiochem. 2019 Feb 13;20(7):891–5. doi: 10.1002/cbic.201800633 (PMC6468270; doi:10.1002/cbic.201800633)
Supplement: Supplementary file 1 — Supplementary [file CBIC-20-891-s001.pdf]

CHEMBIOCHEM

Supporting Information

Recognition of ASF1 by Using Hydrocarbon-Constrained Peptides

May Bakail+,[a, b] Silvia Rodriguez-Marin+,[c, d] Zsófia Hegedüs,[c, d] Marie E. Perrin,[a] Françoise Ochsenbein,*[a] and Andrew J. Wilson*[c, d]

cbic_201800633_sm_miscellaneous_information.pdf

General experimental points for peptide synthesis

All commercial solvents were purchased and used without further purification unless stated otherwise. Commercially available starting materials and reagents were obtained from Sigma-Aldrich, Alfa Aesar or Fisher Scientific. Amino acid derivatives, coupling reagents and resins were purchased from Novabiochem. Purification by column chromatography was carried out using silica gel (40-63 µm mesh size). Analytical thin layer chromatography (TLC) was conducted using Merck 0.2 mm silica gel 60 F_{254} pre-coated aluminium sheets. ^{1}H and ^{13}C NMR spectra were measured using a Bruker DRX 500 series spectrometer. Chemical shifts are expressed as parts per million using solvent as internal standard and coupling constants (*J*) are reported to the nearest 0.1 Hz. The following abbreviations are used: s for singlet, d for doublet, t for triplet, q for quartet and m for multiplet. High resolution mass spectrometry (HRMS) was carried out using a Bruker MicroTOF mass spectrometer or a Bruker Maxis impact mass spectrometer, in both cases under electro-spray ionisation (ESI) conditions. Infra-red spectra were recorded using a Perkin-Elmer Spectrum One FT-IR spectrophotometer. Elemental combustion analyses were performed by the School of Chemistry Microanalysis facility using a Carlo Erba Elemental Analyser MOD 1106 instrument and the found composition is reported to the nearest 0.05%. LC-MS experiments were run on a Bruker Daltonics HTCUltra™ series spectrometer and were run through a C18 column on a methanol/water gradient (0-95% MeCN over 3 minutes). Analytical HPLC experiments were run on an Agilent 1290 Infinity LC series spectrometer. Mass-directed preparative HPLC experiments were run using an Agilent 1260 Infinity Preparative system and analysed by a 6120 Quadrupole detector.

General methods for manual Solid Phase Peptide Synthesis

All reactions were performed at room temperature unless otherwise stated.

Method A: **Resin Swelling**

Rink amide MBHA resin (0.1 mmol) was used for all syntheses unless stated otherwise. The resin was placed in a vaculate reservoir, dichloromethane (3 mL) was added and the resin was agitated on a blood-spinner for 30 min to allow its swelling.

Method B: **Fmoc Deprotection**

N-terminal Fmoc protecting groups were removed by the addition of 20% piperidine in DMF (5 × 2 mL × 2 min), followed by washing of the resin with DMF (5 × 2 mL × 2 min).

Method C: **Kaiser Test**[1]

The Kaiser Test was employed to determine the successful coupling of most of the amino acid residues. A small amount of resin beads was rinsed with dichloromethane and placed in a vial, followed by the addition of two drops of each of the following three solutions in the respective order:

1) Ninhydrin (5% w/v) in ethanol;

2) Phenol (80% w/v) in ethanol;

3) 1 mM KCN(aq.) in pyridine (2% v/v).

The solution was then heated to ca. 100 °C for 1 min. Yellow solution and no change in the colour of the beads indicate successful couplings, whereas blue solution and dark colour of the beads indicate presence of primary amines as a result of incomplete couplings. In the second case, a double coupling was necessary. This colour test can be used to identify free primary amines, however is inconclusive for Asp, Ser, Pro and Asn residues.

Method D: **Chloranil Test**[2]

The chloranil test was also employed to determine successful couplings of some amino acid residues. A small amount of beads was rinsed in dichloromethane and placed in a vial, followed by the addition of two drops of each of the following solutions in the respective order:

1) Acetaldehyde (2% v/v) in DMF;

2) *p*-Chloranil (2% w/v) in DMF.

The solution was left at rt for 5 min. No change in colour of the beads showed successful couplings, whereas the change of bead colour to pale green/bright blue indicate presence of primary amines as a result of incomplete couplings. In the second case, a double coupling was necessary. This test is a reliable method to detect secondary amines; therefore, it was particularly useful for Pro residues.

Method E: **Chain Elongation**

The coupling of the desired amino acids (5 equiv) was performed with HCTU or HATU (5 equiv) and DIPEA (5 equiv) dissolved in DMF (2 mL) and added to the resin, followed by agitation for 1 h (2 h for problematic couplings). For double couplings, this step was repeated. After draining the reagents, the resin was washed with DMF (3 × 2 mL × 2 min) and the success of coupling determined by a negative colour test (*Method C* or *D*). Once a coupling was confirmed successful, the *N*-terminal Fmoc was deprotected (*Method B*) and a subsequent coupling or *N*-terminal acetylation (*Method F*) was performed.

<u>Method F:</u> *N*-terminal Acetylation

After complete peptide chain elongation, *N*-terminal acetylation was performed with acetic anhydride (10 equiv) and DIPEA (10 equiv) dissolved in DMF (2 mL), which was transferred to the resin for 2 h. Later the resin was drained, washed with DMF (3 × 2 mL × 2 min) and successful capping determined by a negative colour test (*Method C* or *D*).

<u>Method G:</u> On-Resin Olefin Metathesis

After *N*-terminal acetylation, on-resin olefin metathesis was performed using a 10 mM solution of Grubbs 1st Generation Catalyst in degassed dichloroethanol (2 mL), which was transferred to the vessel (2 × 2 h).

<u>Method H:</u> Cleavage and Deprotection

After *N*-terminal acetylation (and on-resin olefin metathesis when required), the resin was washed with DMF (5 × 2 mL × 2 min.), dichloromethane (5 × 2 mL × 2 min.), and then Et_2O (3 × 2mL × 2min.). Peptides were then simultaneously cleaved and side chain deprotected with cleavage "Reagent K" TFA : EDT : Thioanisole : Phenol : H_2O, 87:3:5:5:5 (2 × 2 mL × l h). Peptides with large number of Arg(Pbf) residues required incubation times up to 3 h for complete deprotection of the side chains. The resin was washed with fresh TFA (2 mL × 2 min) and the TFA evaporated under N_2 (g). The resulting oil was precipitated with ice-cold ether and placed in a centrifuge (3000 rpm × 2 min). The supernatants were removed, the precipitate rinsed with ice-cold ether (3 × 3000 rpm × 2 min) and dried *in vacuo*.

<u>Method I:</u> Mass – Directed HPLC Purification

Crude peptides were dissolved in either DMSO, 1:1 dioxane:water or 9:1 water:hexafluoroisopropanol at an approximate concentration of 20 mg mL^{-1}. Peptides were purified using reversed phase mass directed HPLC with software Masshunter by ChemStation (Agilent). The columns used were a Jupiter Proteo or an Agilent XBridge 5 µm 19×100 mm C18 preparative column. An increasing gradient of MeCN to water (plus 0.1% formic acid v/v in both solvents) at a flow rate of 20 mL min^{-1} was used as mobile phase; in each case the gradient was optimized to obtain the best separation of the desired peptide from the rest of impurities from the crude mixture. Mass directed chromatography allows the collection of the desired peptide by mass, with the eluent split into an Agilent 6120 Quadrupole LCMS which triggers collection of eluent at a programmed *m/z*. This technique is particularly useful for peptides with weak UV traces. When the separation of the desired peptide from the impurities was problematic, the collection was performed using time slices at the required time interval.

The resulting fractions were checked on the analytical HPLC, concentrated by centrifugal evaporation (Genevac), re-suspended in water and lyophilized.

General methods for automated Solid Phase Peptide Synthesis

Rink amide MBHA resin (0.1 mmol) was used for automated peptide synthesis, with couplings mediated by HCTU (5 equiv) and DIPEA (5 equiv) as base. The synthesis of peptides using the microwave assisted Liberty CEM™ Peptide Synthesiser followed the cycle below:

Resin Loading

Clean reaction vessel; wash with DMF; wash with dichloromethane; transfer resin to reaction vessel; wash with DMF; wash with dichloromethane; transfer resin to reaction vessel; wash with DMF; wash with dichloromethane; vessel draining.

Deprotection and coupling

Clean resin dip tube, wash with DMF (15 mL), add 20% piperidine in DMF (6 mL), agitation at rt (10 min), wash with DMF (15 mL), add 20% piperidine in DMF (6 mL), agitation at rt (10 min), wash with DMF (15 mL), clean resin dip tube, wash with DMF (15 mL), add amino acid (2.5 mL), add coupling reagent (1 mL), add activator base (0.5 mL), agitation at rt (90 min), wash with DMF (15 mL), drain. A standard operating temperature was used for couplings (75°C) After the final residue, the resin is ejected from the reaction vessel, *N*-terminal acetylation and cleavage/deprotection were performed manually (*Methods F* and *H*).

Peptide characterization data

Below are the analytical HPLC and HRMS data for the peptides. The chromatograms correspond to the UV signal obtained at 220 nm in the following conditions: (5-95% MeCN:water and 0.1% TFA v/v in both solvents), t = 4.91 min, 0.5 mL min^{-1}, Ascentis Express C_{18} column. Peptide identity was confirmed by the assessment of multiple charge states, which are tabulated as the *monoisotopic* peak for the Expected and Observed masses. The mass spectra shown below report the *most abundant* isotope peaks. The peptides were dissolved in pure water or DMSO for its characterization.

$H3_{118-135}$ **Ac - GAMGKDIQLARRRIRGERA - $CONH_2$**

$H3_{118-135}$ was obtained using the general methods for SPPS to give 11 mg (11% yield), > 97 % purity.

Analytical HPLC data for **$H3_{118-135}$**

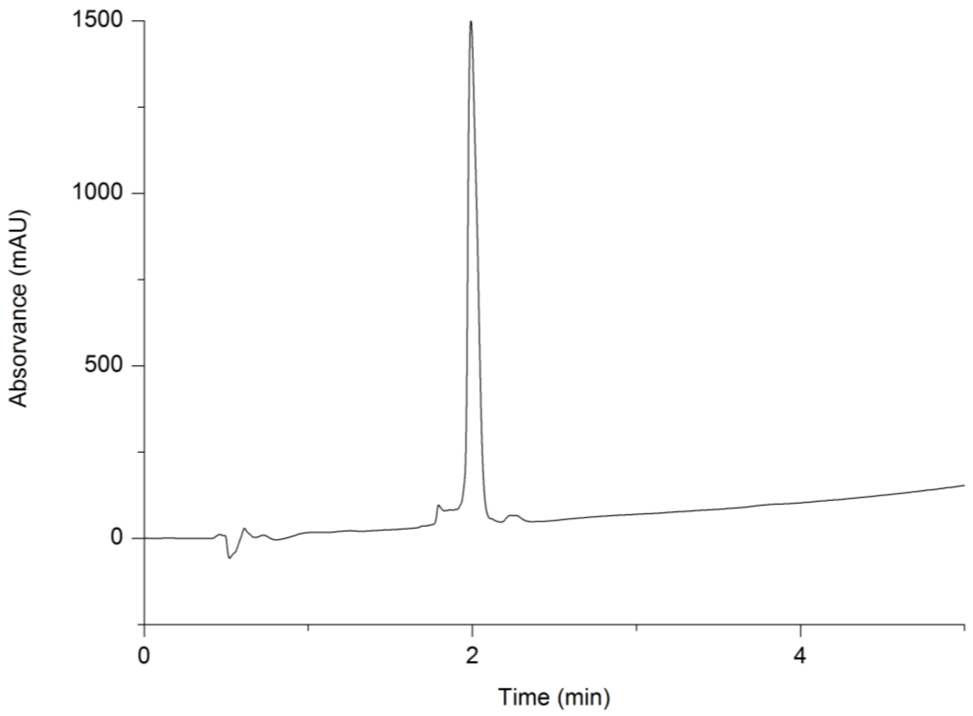

HRMS data for **$H3_{118-135}$**

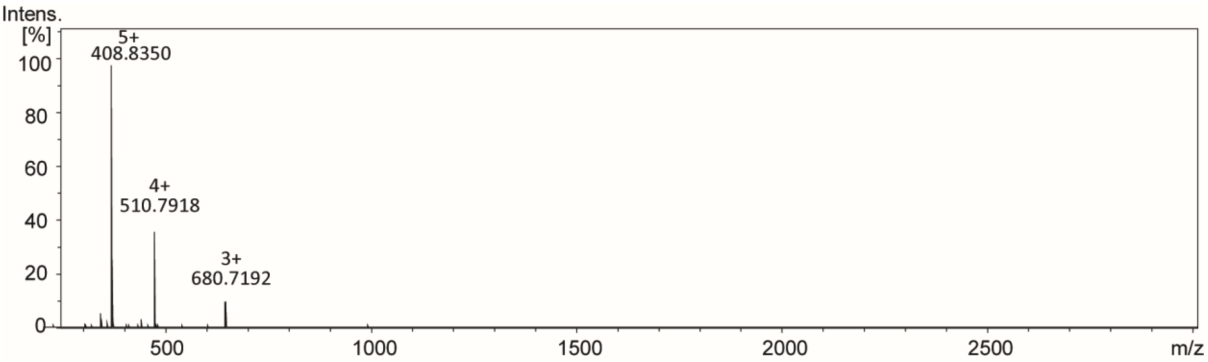

$H3_{118-135}$	Observed	Expected
$[M+3H]^{3+}$	680.3848	680.3850
$[M+4H]^{4+}$	510.5413	510.5405
$[M+5H]^{5+}$	408.6344	408.6339

H3$_{118-135(St120-124)}$

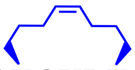

Ac - GAXGKDXQLARRIRGERA - CONH$_2$

H3$_{118-135(St120-124)}$ was obtained using the general methods for SPPS to give 10 mg (5% yield), > 90 % purity.

***Analytical HPLC data for* H3$_{118-135(St120-124)}$**

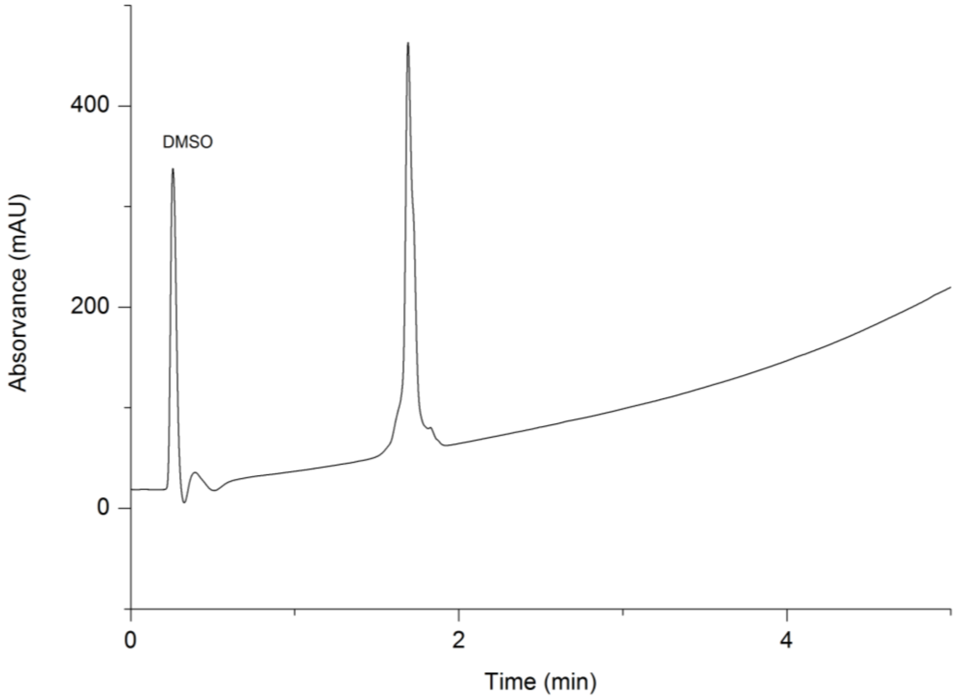

***HRMS data for* H3$_{118-135(St120-124)}$**

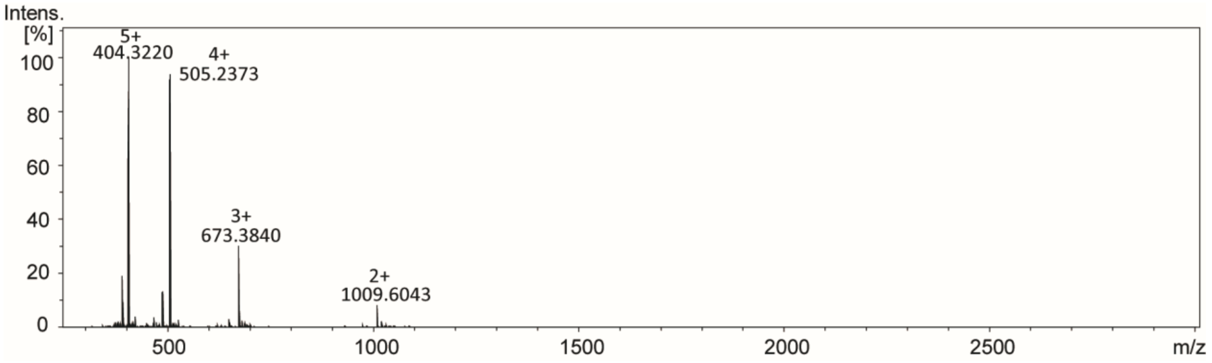

H3$_{118-135(St120-124)}$	Observed	Expected
$[M+2H]^{2+}$	1009.1023	1009.0799
$[M+3H]^{3+}$	673.0500	673.0557
$[M+4H]^{4+}$	504.9862	505.0436
$[M+5H]^{5+}$	404.1218	404.2363

H3$_{118\text{-}135(St123\text{-}127)}$GCA

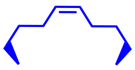

Ac - GAMGKXIQLXRRIRGERAGCA - CONH$_2$

H3$_{118\text{-}135(St123\text{-}127)}$GCA was obtained using the general methods for SPPS to give 6 mg (3% yield), > 90% purity

***Analytical HPLC data for* H3$_{118\text{-}135(St123\text{-}127)}$GCA**

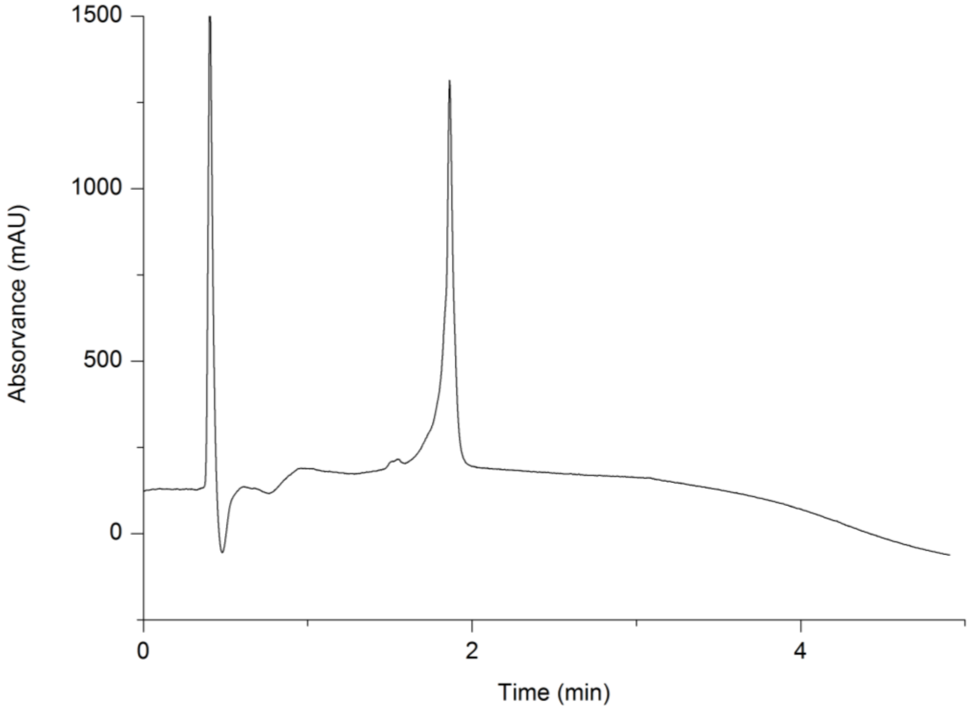

***HRMS data for* H3$_{118\text{-}135(St123\text{-}127)}$GCA**

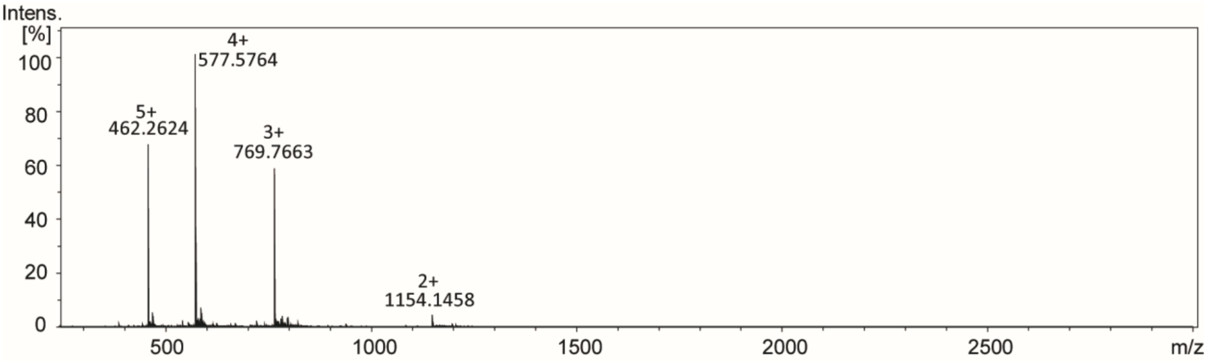

**H3$_{118\text{-}135(St123\text{-}127)}$GCA**	**Observed**	**Expected**
$[M+2H]^{2+}$	1153.6446	1153.6441
$[M+3H]^{3+}$	769.4320	769.4318
$[M+4H]^{4+}$	577.3255	577.3257
$[M+5H]^{5+}$	462.0613	462.0620

Circular Dichroism

Circular Dichroism was performed on an Applied Photophysics ChiraScan Apparatus and Software. For each scan, the following parameters were used: 180-260 nm range; point time 1 s; 1 nm per point; step = 1; bandwidth 4.3 nm; path length 1 mm; temperature 20 °C. Scans were done in duplicate. Samples were dissolved in 40 mM sodium phosphate buffer pH 7.50 at concentration of 100 µM.

$$[\theta] = \frac{\theta}{10 \times c \times l}$$

$$[\theta]_{MRE} = \frac{[\theta]}{(R-1)}$$

Where θ = circular dichroism at a given wavelength, *c* = molar concentration, l = path length in cm, R = number of residues in the peptide sequence.

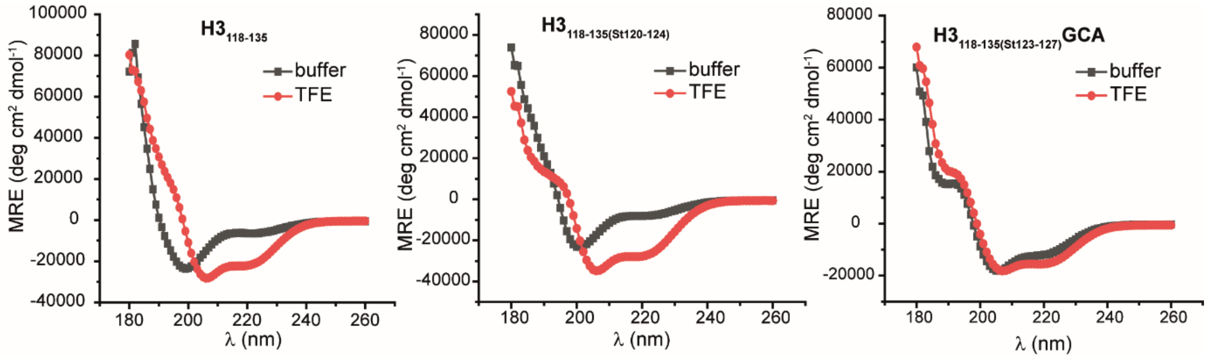

Figure ESI 1. Conformational analyses of histone H3 variant peptides by circular dichroism (CD) analyses (peptide concentration =100 µM, 40 mM sodium phosphate (or 40 mM sodium phosphate/ 30% TFE), 293K, pH7.5)

Expression and Purification of ASF-1

Recombinant human ASF1 (ASF1A(1-156)) was purified as already described from expression in E. coli of a (His)6–GST –Tev site–Asf1 fusion protein using the pETM30 plasmid. [3] Briefly, soluble (His)6-tagged GST fusion protein was purified on reduced glutathione (GSH) agarose beads (Sigma). After cleavage with recombinant (His)6-TEV protease at room temperature overnight, the (His)6-GST tag and the protease were trapped in a Ni-NTA agarose column (Qiagen). The flow-through fraction containing ASF1 proteins was further purified by anion exchange chromatography using a Resource Q 6mL column (GE Healthcare). ASF1 was then concentrated using an Amicon device (Millipore) and the buffer was replaced with a 50mM Tris-HCl (pH7.5). Unlabled ASF1 used for ITC experiments was purified from pellets of bacteria grown in LB medium, and uniformly labelled ASF1 from bacteria gown in M9 minimal media supplemented with (^{15}NH4)Cl (Eurisotop,0.5 g.l−1) as the sole nitrogen source.

NMR studies – ^{1}H-^{15}N HSQC

The binding mode of the constrained peptides was assessed using NMR spectroscopy. NMR experiments were performed at 298°K on Bruker Avance II-600MHz or Bruker Neo-700MHz spectrometers equipped with a TCI cryoprobes. Purified uniformly ^{15}N labelled ASF1A(1-156) was concentrated to 40µM and exchanged in NMR buffer (50mM Tris-HCl pH7.5, 0.1mM EDTA, 0.1mM DSS, 0.1mM NaN_3, protease inhibitor cocktail (at the concentration recommended by the provider, Roche), 10% D_2O), 5mM of TCEP was added for the titration with **$H3_{118\text{-}135(St123\text{-}127)}$GCA** to prevent formation of S-S bridges of the peptide Cys residue. A ^{1}H-^{15}N HSQC reference spectrum was recorded before addition of the peptides (Green spectra, **Figure ESI 2A)**. Proton chemical shifts (in ppm) were referenced relative to internal DSS and ^{15}N reference was set indirectly relative to DSS using frequency ratios (Wishart et al., 1995). NMR data were processed using Topspin (Bruker) and analyzed using Sparky (T.D. Goddard and D.G. Kneller, UCSF). Amide assignment were taken from.[3] The titration experiments were done by adding increasing amounts of concentrated peptide to sample. At each ASF1:peptide ratio, a two dimensional ^{1}H-^{15}N sofast HMQC spectrum was recorded. At a final concentration ratio ASF1:peptide of 1:2 a ^{1}H-^{15}N HSQC reference spectrum was recorded (**Figure ESI 2A)**. Changes in chemical shift and intensities were measured for all resonances for all recorded spectra. Chemical shift variation was calculated with the following formula $\Delta\delta = [(\delta_{HN}{}^{b}-\delta_{HN}{}^{f})^2 + (0.17(\delta_{N}{}^{b}-\delta_{N}{}^{f}))^2]^{1/2}$, where b and f refer to the bound and free form respectively (**Figure ESI 2B)**.

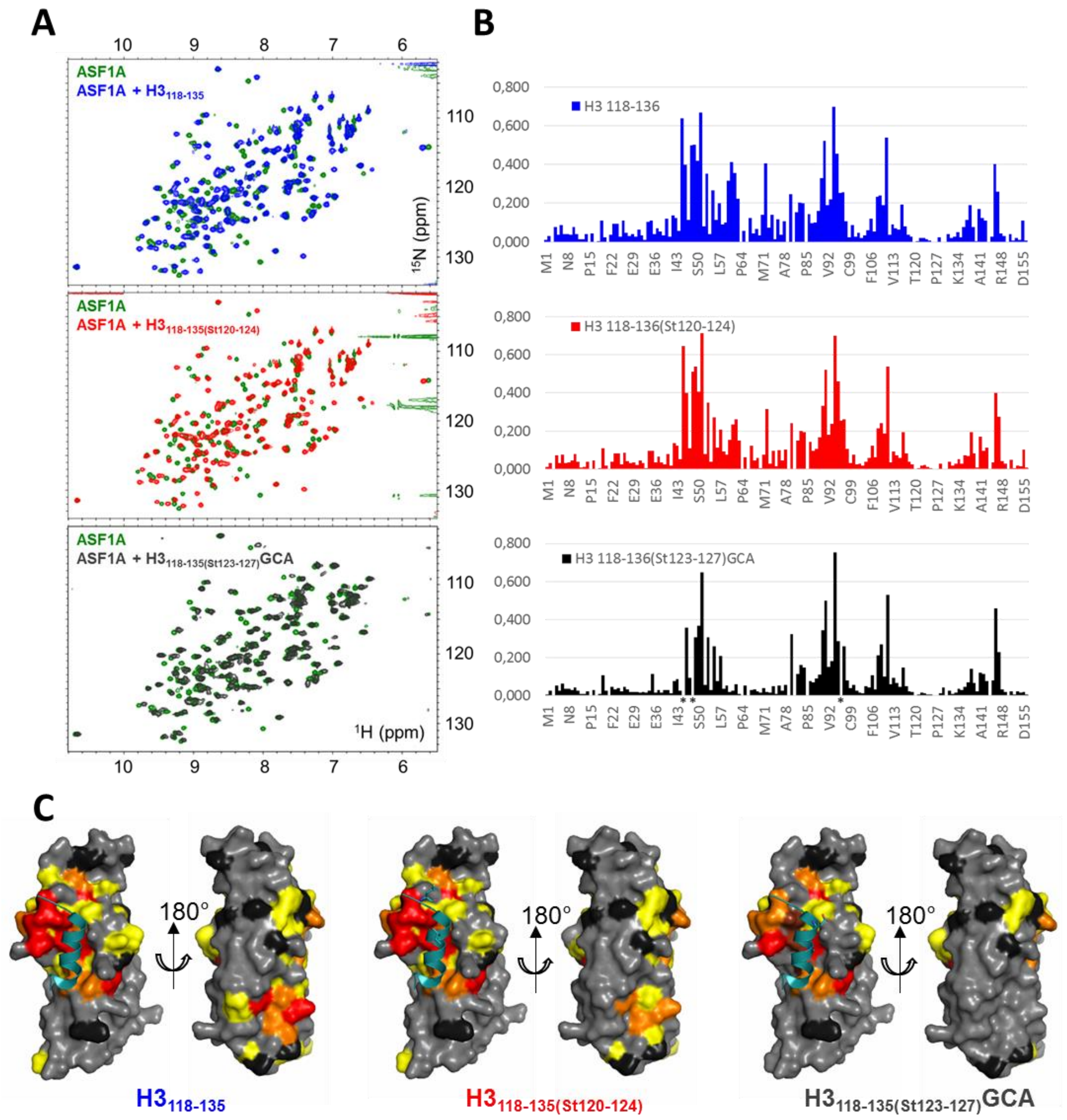

Figure ESI 2. A) ^{1}H-^{15}N HSQC spectra of ASF1A(1-156) alone (green) and after addition of the indicated peptide at a protein:peptide ratio of 1:2. B) Chemical shift perturbation after addition of an excess of the indicated peptide. Stars indicate the disappearance of the signal due to chemical exchange. C) mapping of the chemical shift variation after addition of an excess of the indicated peptide onto the structure of ASF1A(1-156)-H3 (PDB ID: 2IIJ) highlighting the shift changes of the residues with the following color code: red $\Delta\delta>0.4$, orange $0.4<\Delta\delta<0.2$, yellow $0.2<\Delta\delta<0.1$, grey $\Delta\delta<0.1$, relative to free ASF1 spectra, maroon disappearing of the signal after addition of the peptide. The *C*-terminal H3 peptide is represented in blue and the position of residues used for the stapling are shown as sticks.

Isothermal Titration Calorimetry

All IsoThermal Calorimetry (ITC) experiments were performed in a VP-ITC titration calorimeter (Microcal/Malvern) at 20°C, in a 50mM Tris-HCl pH 7.5 buffer. Protein and peptides concentrations were set to values 10µM and 200µM, respectively. Protein and peptide samples were prepared in the same buffer and degassed (ThermoVac, Malvern). After equilibrating the cell at 298°K, the rotating syringe (310rpm) injected at intervals of 280s, 6µL aliquots of peptide solution into the Asf1 previously introduced in the sample cell until saturation was observed. Raw ITC data were processed with the Origin 7.0 Software (OriginLab, Malvern) using the One-Set of Sites fitting model. All ITC experiments were performed at least in duplicate.

Proteolysis assay

Proteolytic stability of peptides **$H3_{118\text{-}135}$**, **$H3_{118\text{-}135(St120\text{-}124)}$** and **$H3_{118\text{-}135(St123\text{-}127)}$GCA** was assessed against trypsin and proteinase K. Experiments were performed in a 96-well plate at room temperature. The experiment wells contained aqueous buffer (50 mM Tris, pH 8, 100 mM NaCl, 170 µL) to which peptide solution (10 µL, 1 mM in H_2O) and enyzme solution (20 µL 0.5 µg/ml trypsine or 20 µL 1 µg/ml proteinase K) was added. Final concentrations in the experiment wells were 50 µM peptide, 0.05 µg/ml trypsine or 0.1 µg/ml proteinase K. A control experiment was started at the same time without the enzymes. 10 µL aliqouts of the reaction were quenched at different time-points using 90 µL 2% TFA solution. Samples were measured using LC-ESI-MS on a Bruker Maxis impact mass spectrometer equipped with a Thermo Ultimate 3000 UPLC. All samples were run on an Acquity BEH C4 column using a gradient of 1-90% B (A: H_2O + 0.1 % formic acid, B: ACN + 0.1 % formic acid) over 4 minutes. Intact peptide content was determined relative to the t=0 time-point based on HPLC peak areas. Curves were fitted using an exponential decay function in Origin Pro 9.

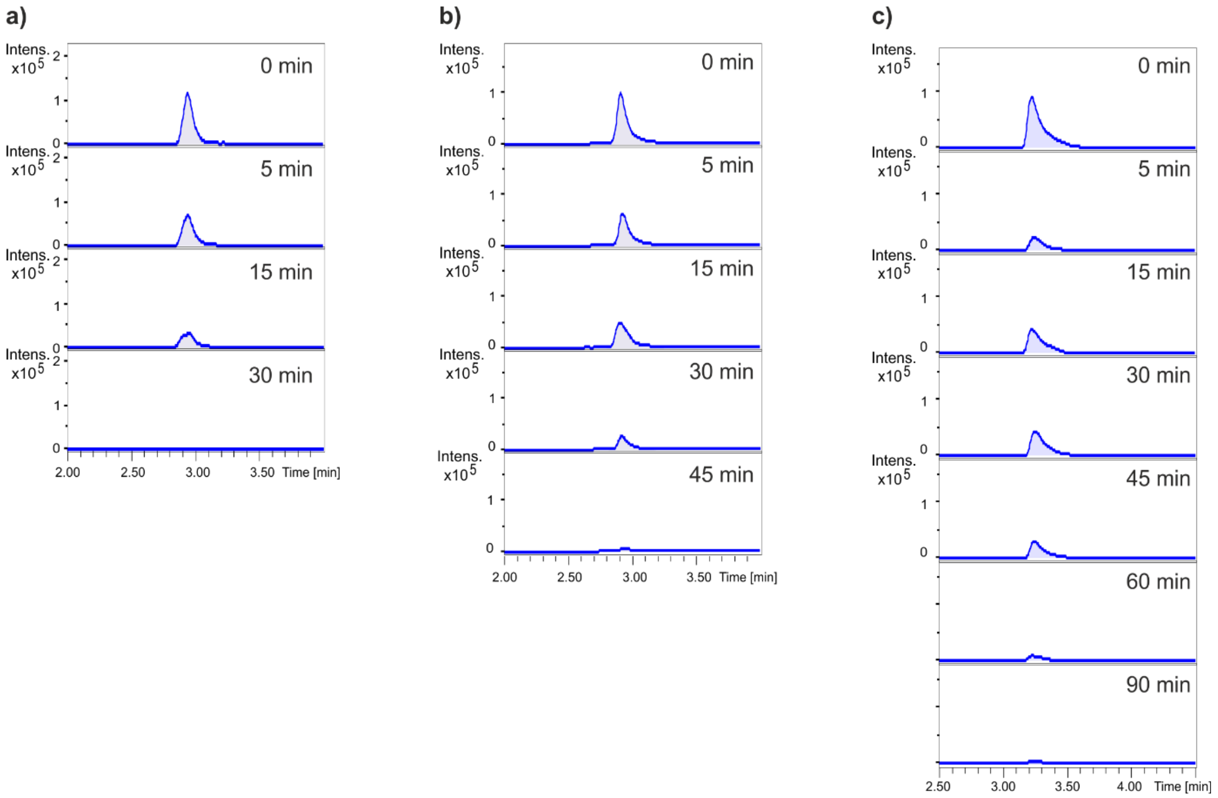

Figure ESI3. Extracted ion chromatograms at different time-points cleaved by trypsin **a) H3$_{118\text{-}135}$ b) H3$_{118\text{-}135(St120\text{-}124)}$ c) H3$_{118\text{-}135(St123\text{-}127)}$GCA**

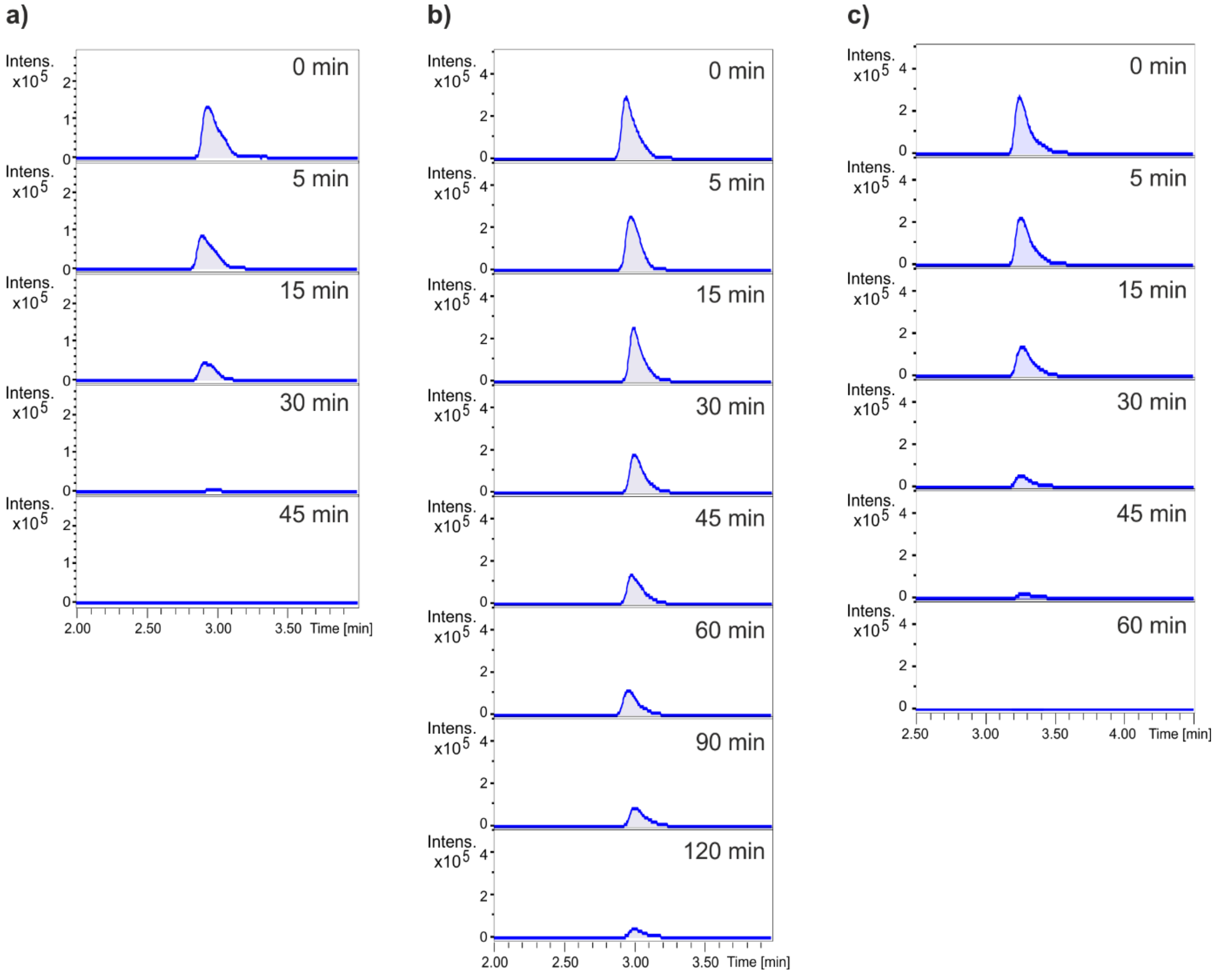

Figure ESI4. Extracted ion chromatograms at different time-points cleaved by proteinase K **a) H3$_{118\text{-}135}$ b) H3$_{118\text{-}135(St120\text{-}124)}$ c) H3$_{118\text{-}135(St123\text{-}127)}$GCA**

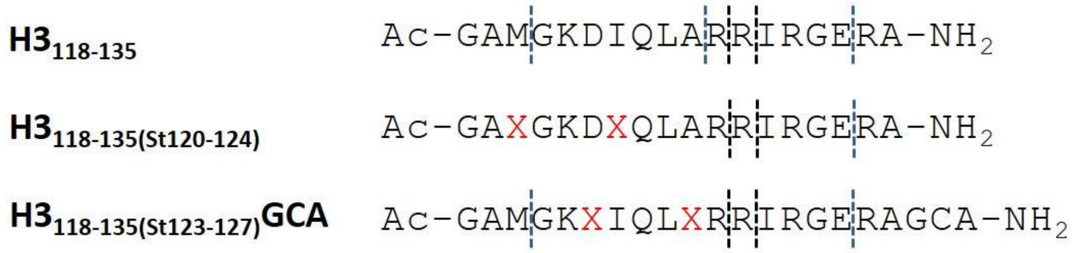

Figure ESI5. Identified cleavage sites for all peptides, proteinase K clevage site marked by blue dahsed lines, trypsine cleavage sites marked by black dahsed lines. Cleavage occurs at C-terminal direction of the marked amino acid.

References

[1] E. Kaiser, Colescot.Rl, Bossinge.Cd, P. I. Cook, *Anal. Biochem.* **1970**, *34*, 595.
[2] T. Vojkovsky, *Pept. Res.* **1995**, *8*, 236.
[3] F. Mousson, J. Couprie, J.-Y. Thuret, J.-M. Neumann, C. Mann, F. Ochsenbein, *J. Biomol. NMR* **2004**, *29*, 413.
